# Supplementary figures and images for: Syntaxin 17 recruitment to mature autophagosomes is temporally regulated by PI4P accumulation
Source: eLife. 2024 Jun 4;12:RP92189. doi: 10.7554/eLife.92189 (PMC11152571; doi:10.7554/eLife.92189)

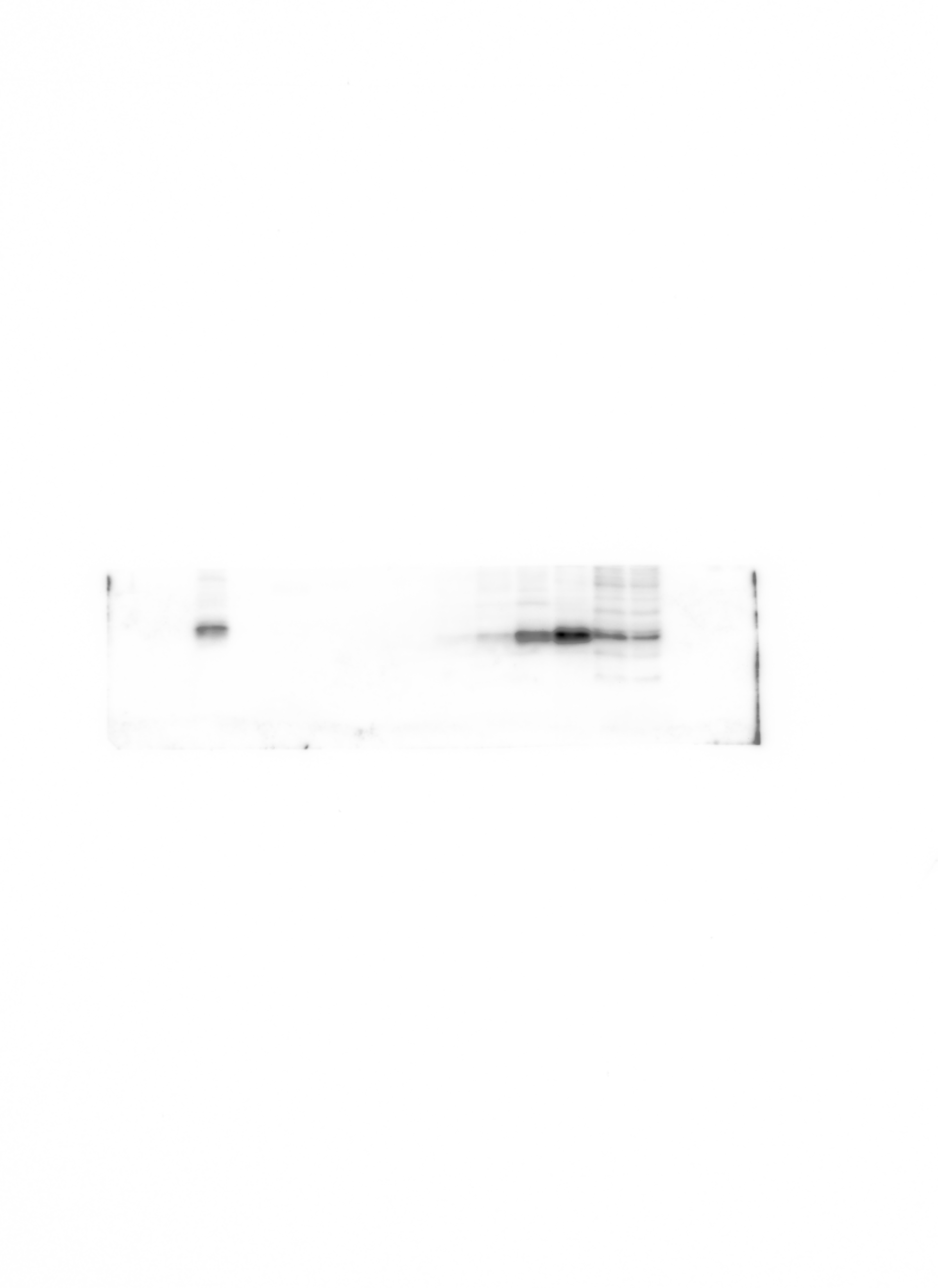

Supplement: Figure 4—source data 2. [file elife-92189-fig4-data2.zip › Figure 4/Figure4B_TOMM20_raw.tif]

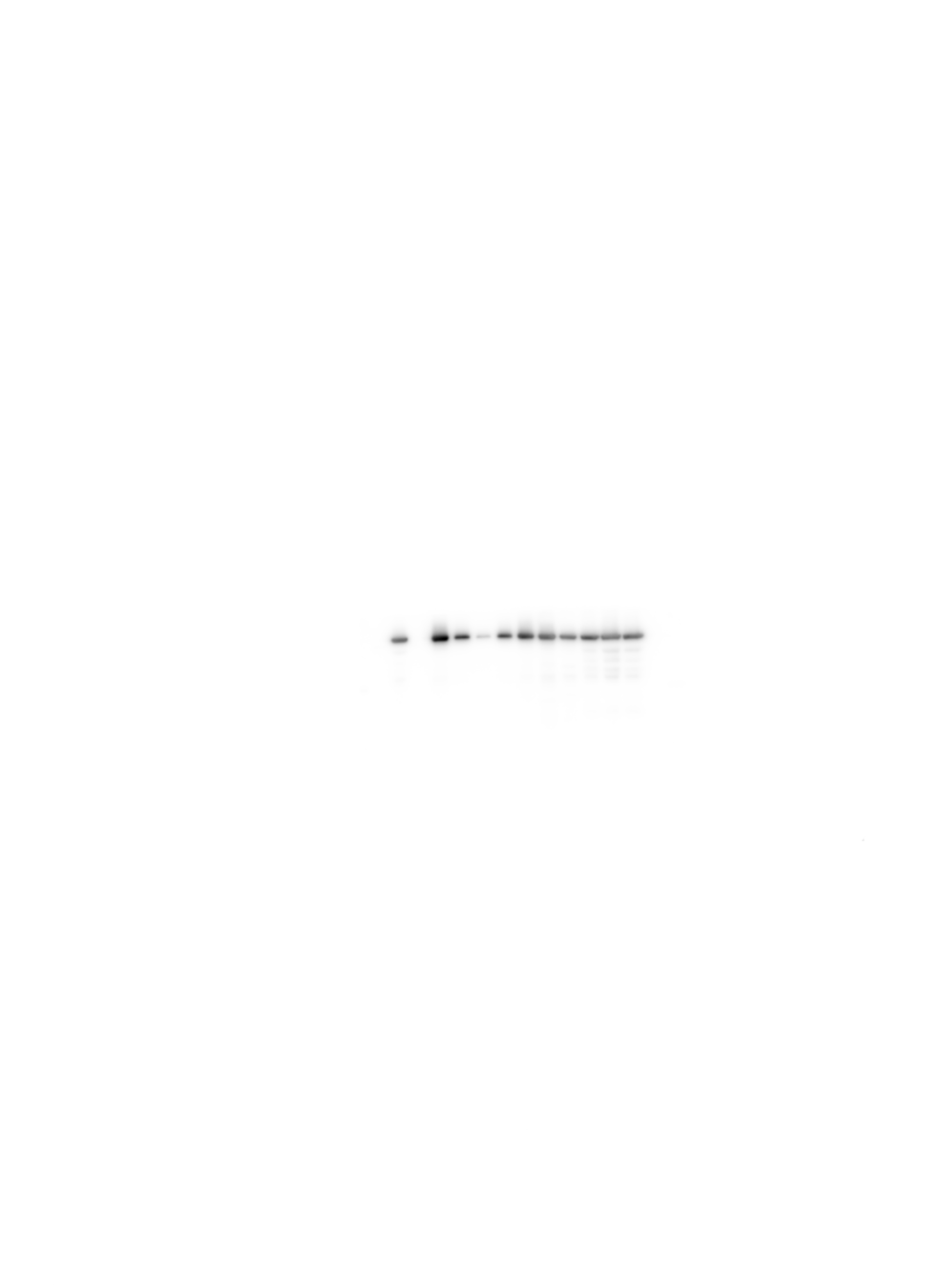

Supplement: Figure 4—source data 2. [file elife-92189-fig4-data2.zip › Figure 4/Figure4B_p62_raw.tif]

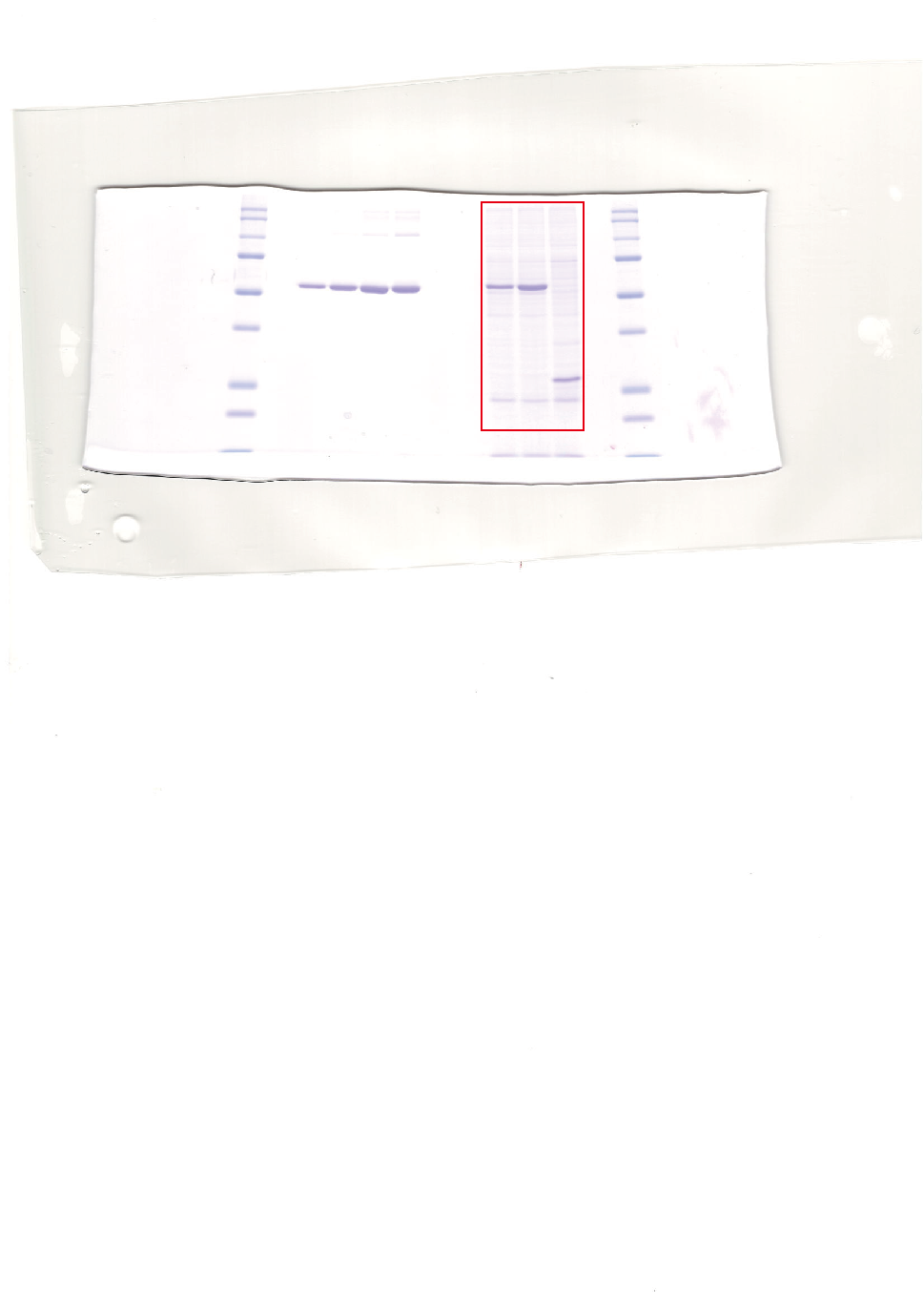

Supplement: Figure 4—source data 2. [file elife-92189-fig4-data2.zip › Figure 4/Figure4C_annotated.tif]

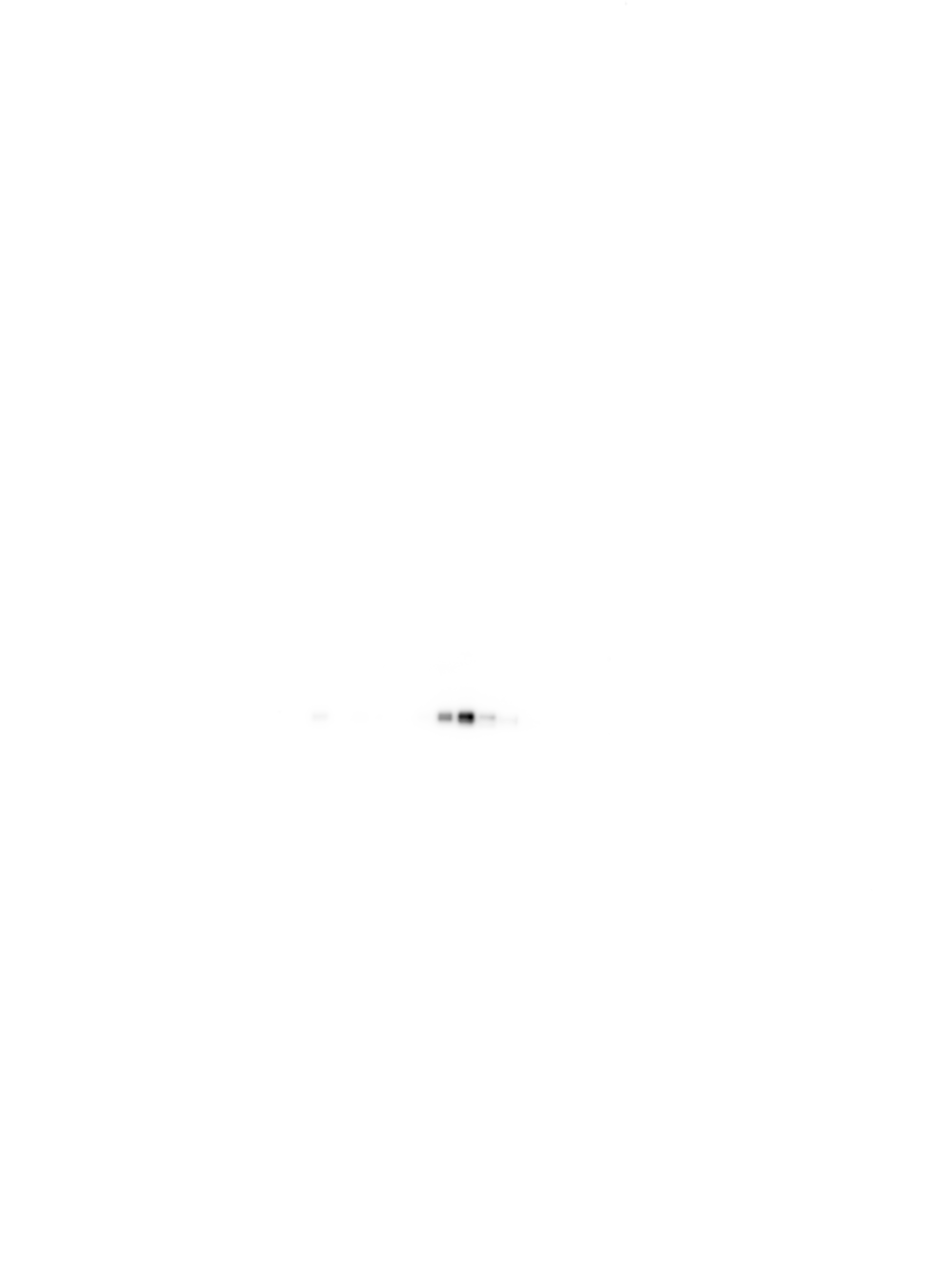

Supplement: Figure 4—source data 2. [file elife-92189-fig4-data2.zip › Figure 4/Figure4B_LAMP1_raw.tif]

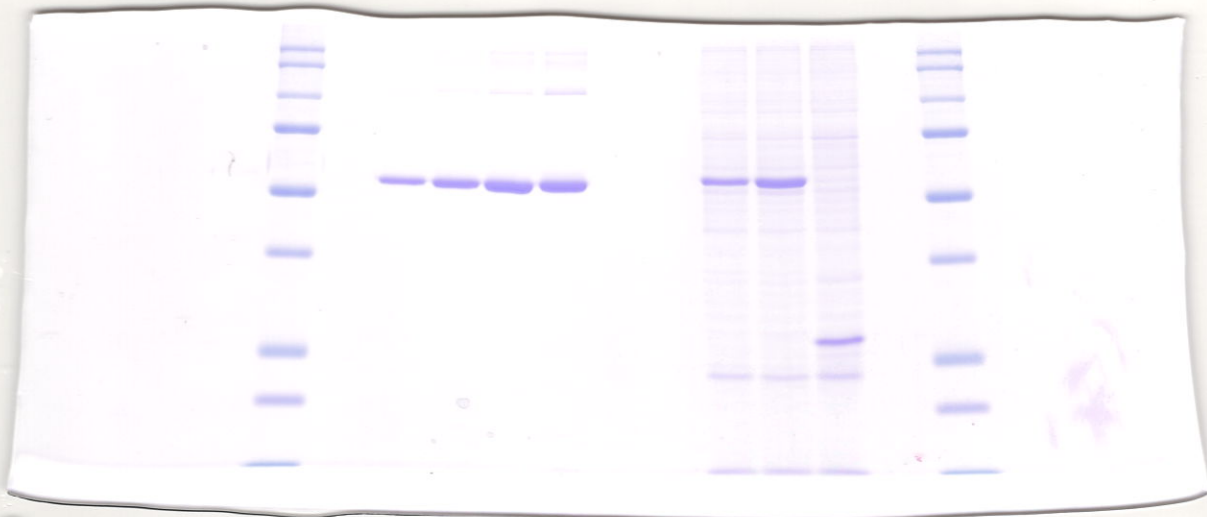

Supplement: Figure 4—source data 2. [file elife-92189-fig4-data2.zip › Figure 4/Figure4C_raw.PDF]

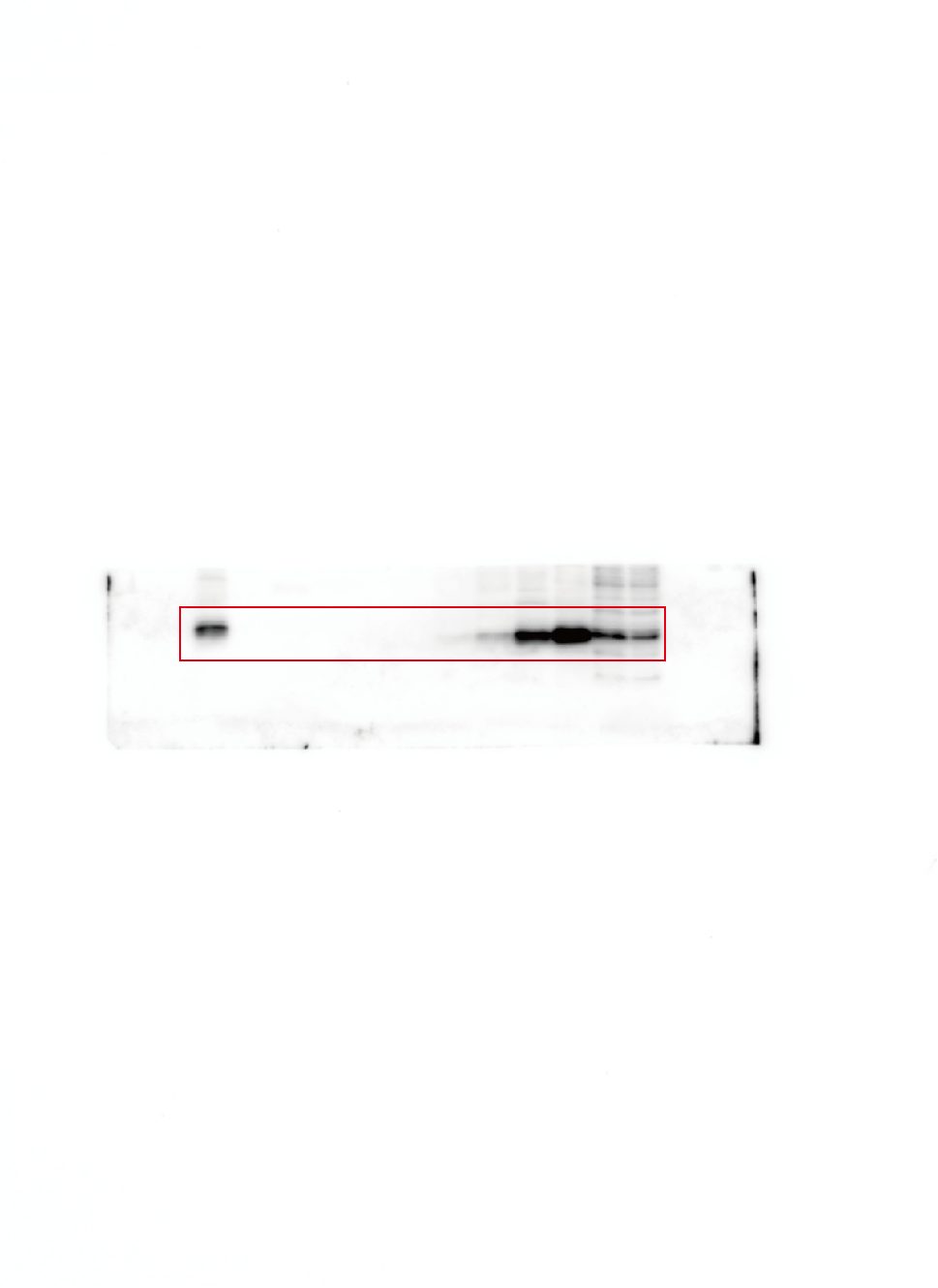

Supplement: Figure 4—source data 2. [file elife-92189-fig4-data2.zip › Figure 4/Figure4B_TOMM20_annotated.tif]

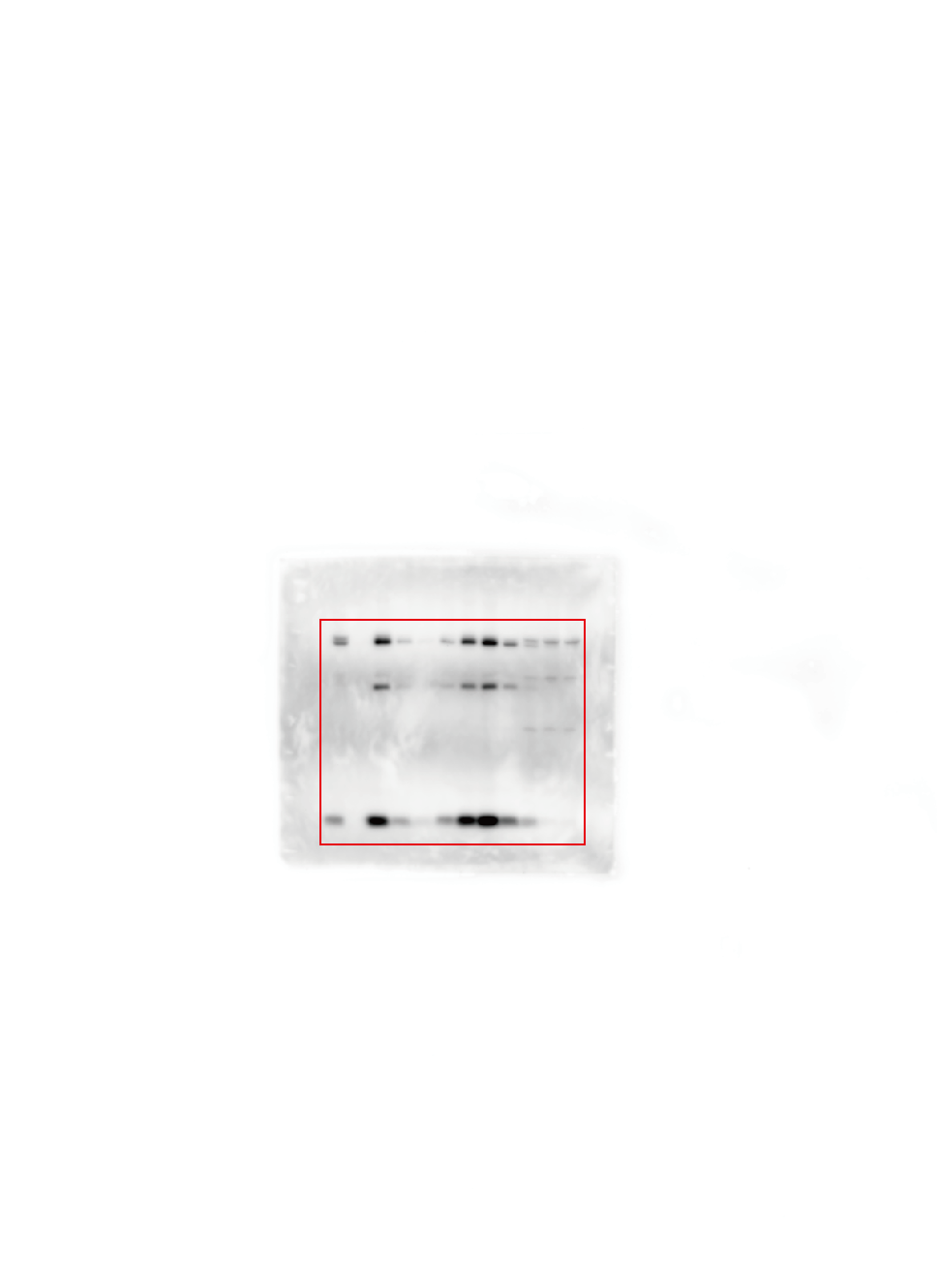

Supplement: Figure 4—source data 2. [file elife-92189-fig4-data2.zip › Figure 4/Figure4B_LC3_annotated.tif]

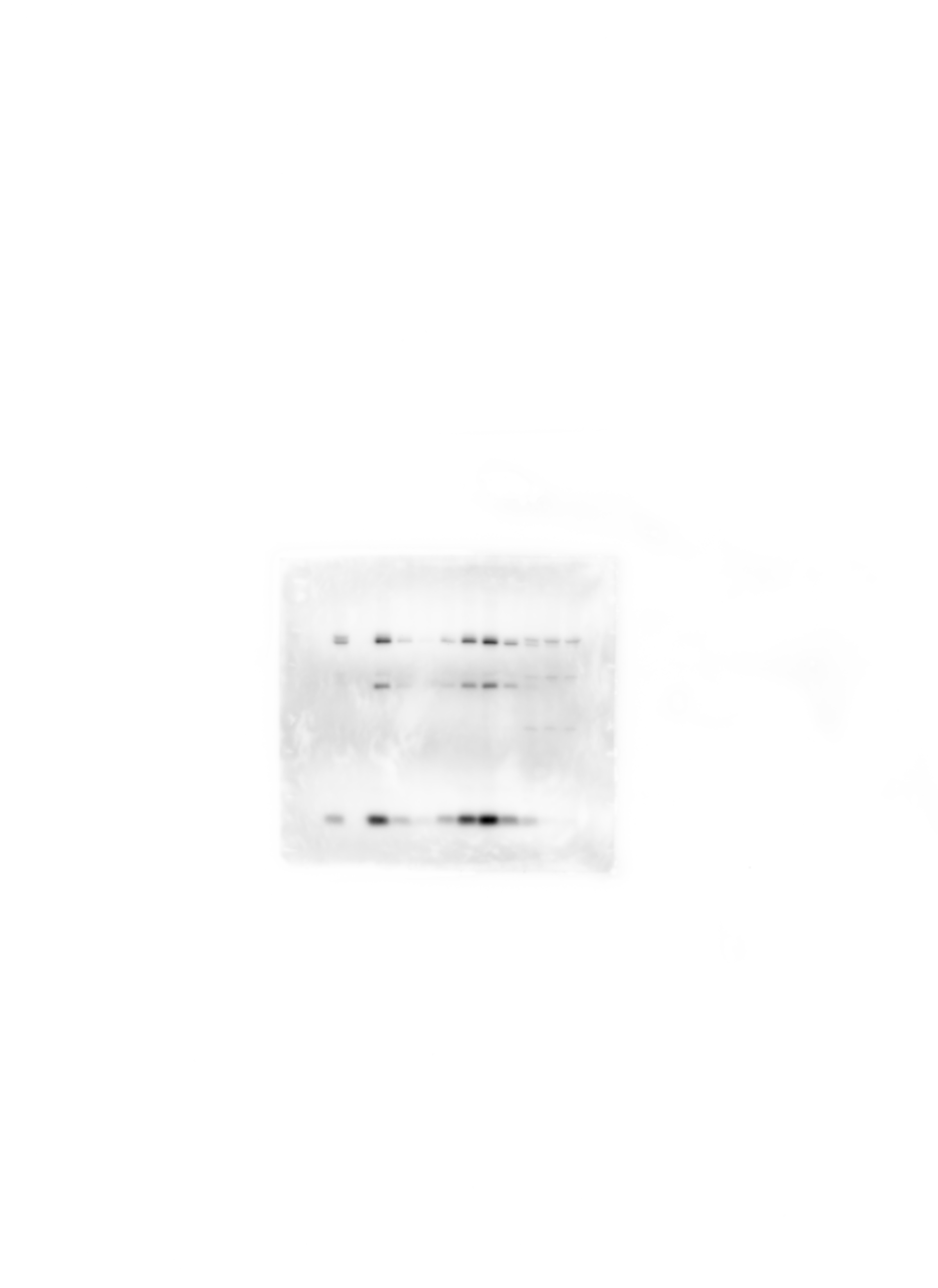

Supplement: Figure 4—source data 2. [file elife-92189-fig4-data2.zip › Figure 4/Figure4B_LC3_raw.tif]

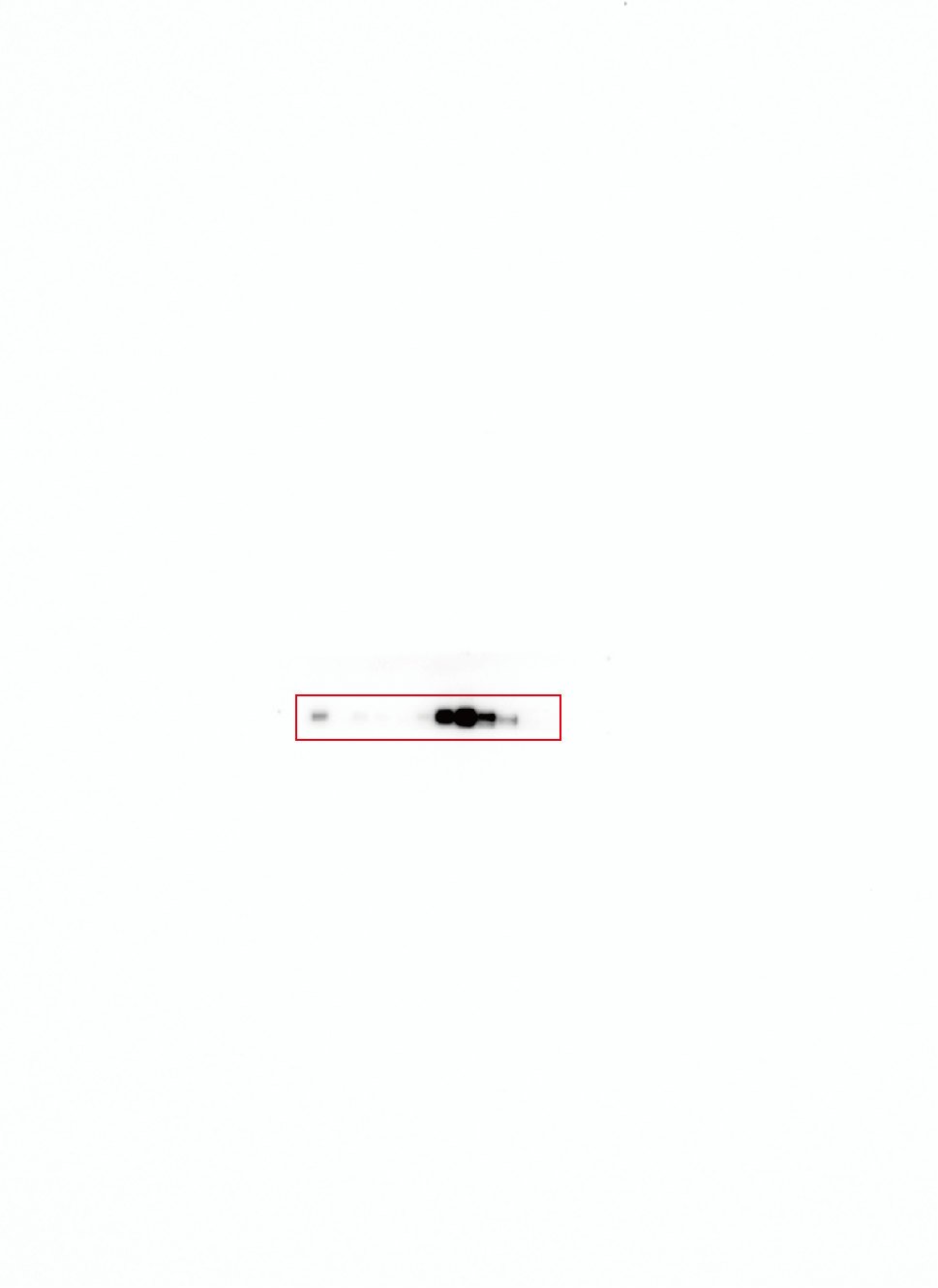

Supplement: Figure 4—source data 2. [file elife-92189-fig4-data2.zip › Figure 4/Figure4B_LAMP1_annotated.tif]

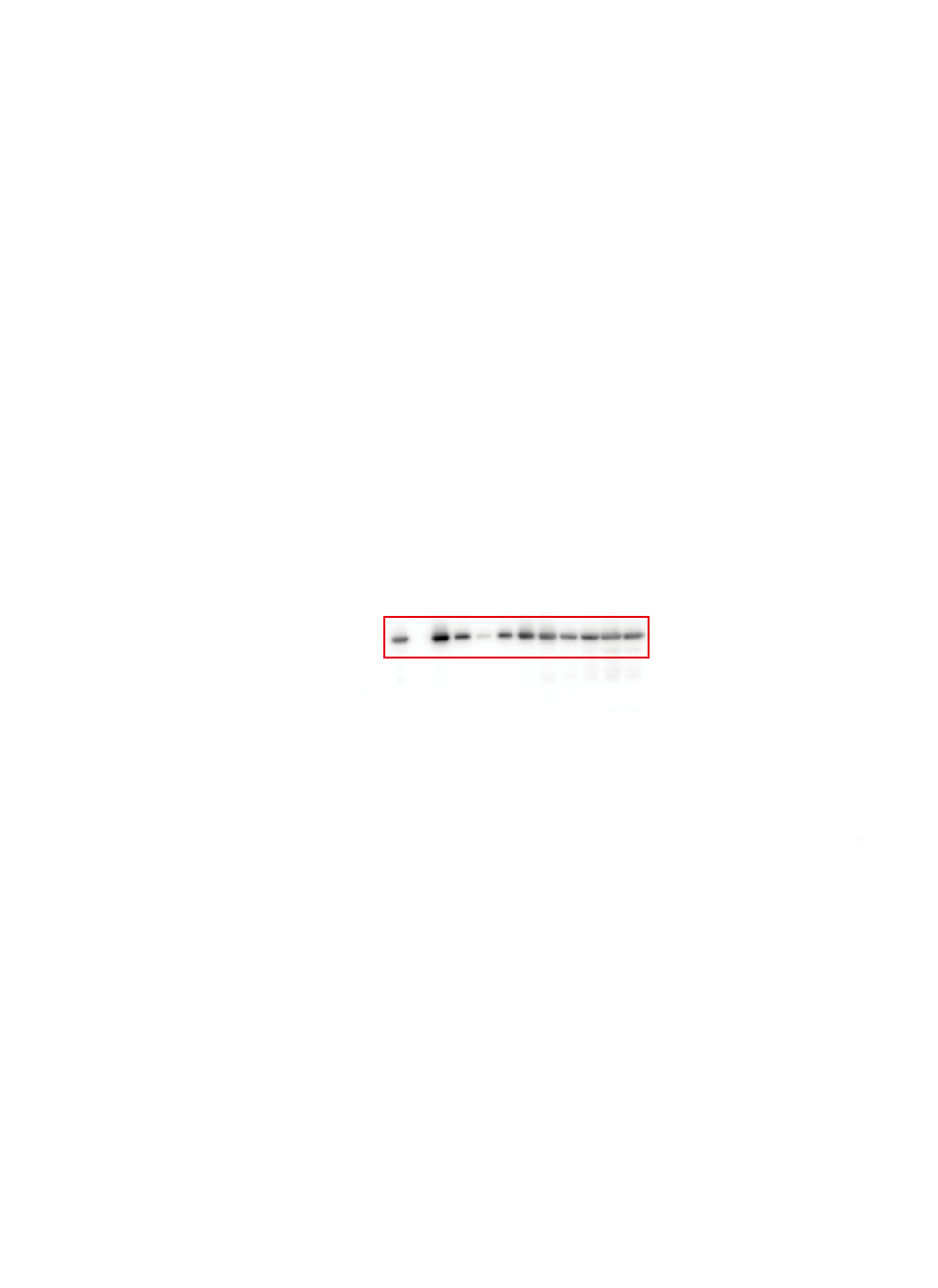

Supplement: Figure 4—source data 2. [file elife-92189-fig4-data2.zip › Figure 4/Figure4B_p62_annotated.tif]
